# Complete genome sequence of bacteriophage Aero_H33 targeting the aquaculture pathogen *Aeromonas jandaei*

**DOI:** 10.1128/mra.00759-26

**Published:** 2026-10-01

**Authors:** Sumitra Sivaprakasam, Heama Maleini Balasubramaniam, Fong Tze Yan, Loh Michelle JiaMin, Marina Hassan Mohd., Yukgehnaish Kumarasan, Sasireigga Jaya Jothi, Kasi Marimuthu, Sivachandran Parimannan, Heera Rajandas

**Affiliations:** 1 Centre of Excellence for Omics-Driven Computational Biodiscovery (COMBio), AIMST University, Bedong, Kedah, Malaysia; 2 Higher Institution Centre of Excellence (HICoE), Institute of Tropical Aquaculture and Fisheries, Universiti Malaysia Terengganu, Kuala Nerus, Terengganu, Malaysia; 3 School of General and Foundation Studies, AIMST University, Kedah, Malaysia; 4 Department of Environmental Science, Tezpur Central University, Tezpur, Assam, India; 5 Center for Evolutionary Hologenomics, Globe Institute, University of Copenhagen, Copenhagen, Denmark; Montana State University, Bozeman, Montana, USA

**Keywords:** *Aeromonas jandaei*, bacteriophage, genome

## Abstract

Bacteriophage Aero_H33, targeting the aquaculture pathogen *Aeromonas jandaei*, was isolated from a seawater sample collected from Penang, Malaysia. The Aero_H33 genome is 38,953 bp in length, contains 57 predicted coding sequences, and shows 96.18% nucleotide identity with 98% genome coverage to Aeromonas phage PZL-Ah1.

## ANNOUNCEMENT

*Aeromonas jandaei* is a gram-negative bacterium that has been associated with significant mortality in farmed freshwater fish, resulting in substantial economic losses (1). As the widespread use of antibiotics for managing bacterial infections in aquaculture has contributed to the increased prevalence of antimicrobial resistance, sustainable alternatives are urgently needed (2, 3). Bacteriophages offer a promising targeted approach for controlling bacterial pathogens. Here, we report the genomic characteristics of a lytic bacteriophage Aero_H33 isolated against *A. jandaei*.

The host bacterium, *A. jandaei* strain H33, was obtained from the Institute of Tropical Aquaculture and Fisheries (AKUATROP), University Malaysia Terengganu. Bacteriophage Aero_H33 was isolated from a seawater sample collected from Penang Island, Malaysia (5.2265873°N, 100.4504672°E). Ten milliliters of 0.22 μm filtered seawater sample was mixed with 10 mL of sterile tryptic soy broth supplemented with 50 μL of CaCl_2_ and 1 mL of overnight culture of strain H33, followed by incubation at 37°C for 24 h with shaking at 180 rpm (4). The resulting lysate was centrifuged at 8,000× *g* for 5 min at room temperature and filtered through a 0.22 µm membrane. Lytic activity was confirmed by spot assay prior to seven cycles of clonal purification (5 ). Aero_H33 exhibited a narrow host range, infecting only the original host strain among the five Aeromonas strains tested.

Genomic DNA was extracted from high-titer Aero_H33 phage lysate using the phenol–chloroform method. Briefly, 500 µL of lysate was treated with CTAB lysis buffer (2 mg of CTAB, 50 µL of 1 M Tris-HCl pH 8, 300 µL of 2.5 M NaCl, 5 µL of 0.5 M EDTA), 10 µL of Proteinase K (20 mg/mL), and 5 µL of β-mercaptoethanol for 1 h at 56°C. Following purification using the phenol–chloroform method (6), nucleic acid concentration was quantified using a Qubit 4 Fluorometer, and the genome was sequenced using the Illumina NovaSeq 6000 platform with the Rapid Plus DNA Library Prep Kit (ABclonal, USA) with a 2 × 150 bp read length.

A total of 5,704,875 reads were obtained, with an average sequencing depth of 378×. Reads were quality-checked using FastQC v0.11.9, trimmed using Trimmomatic v0.39, and subsampled to 100,000 reads using seqtk (7–9). Processed reads were assembled with SPAdes v3.13.1 under default settings, yielding 371× genome coverage. Genome completeness was assessed with CheckV v1.0.1 (10, 11). The genome was subsequently annotated with Pharokka v1.7.1, and termini were predicted using PhageTerm v1.0.12 (12, 13). PhageLeads was then used to assess for therapeutic suitability of the genome (14).

The assembled genome is 38,953 bp in length, with a GC content of 53.48%. Aero_H33 belongs to the family *Autographiviridae* and the genus *Teseptimavirus*. PhageTerm indicated fixed termini with short direct terminal repeats, and CheckV confirmed 100% genome completeness. BLAST analysis showed that the genome is closely related to Aeromonas phage PZL-Ah1 (MT681669.1), and average nucleotide identity analysis with EzBioCloud indicated 96.21% identity (15). Annotation with Pharokka predicted 57 CDS, of which 24 were putative, and no tRNAs. PhageLeads screening detected no genes associated with lysogeny, antimicrobial resistance, or virulence, indicating a lytic lifecycle and potential suitability for therapeutic applications.

## Data Availability

The complete genome sequence of bacteriophage Aero_H33 has been deposited in GenBank under the accession number PZ297965.1, while the raw Illumina reads have been deposited in the Sequence Read Archive under accession number SRR38004817.
